# Systemic Delivery of MicroRNA Using Recombinant Adeno-associated Virus Serotype 9 to Treat Neuromuscular Diseases in Rodents

**DOI:** 10.3791/55724

**Published:** 2018-08-10

**Authors:** Naemeh Pourshafie, Philip R. Lee, Ke-lian Chen, George G. Harmison, Laura C. Bott, Kenneth H. Fischbeck, Carlo Rinaldi

**Affiliations:** ^1^Neurogenetics Branch, National Institute of Neurological Disorders and Stroke, National Institutes of Health; ^2^Section on Nervous System Development and Plasticity, The Eunice Kennedy Shriver National Institute of Child and Human Development, National Institutes of Health; ^3^Department of Molecular Biosciences, Rice Institute for Biomedical Research, Northwestern University; ^4^Department of Physiology, Anatomy and Genetics, University of Oxford

**Keywords:** Genetics, Issue 138, miRNA, RNA therapeutics, rAAV, Gene therapy, Gene silencing, Neuromuscular Disease

## Abstract

RNA interference via the endogenous miRNA pathway regulates gene expression by controlling protein synthesis through post-transcriptional gene silencing. In recent years, miRNA-mediated gene regulation has shown potential for treatment of neurological disorders caused by a toxic gain of function mechanism. However, efficient delivery to target tissues has limited its application. Here we used a transgenic mouse model for spinal and bulbar muscular atrophy (SBMA), a neuromuscular disease caused by polyglutamine expansion in the androgen receptor (AR), to test gene silencing by a newly identified AR-targeting miRNA, miR-298. We overexpressed miR-298 using a recombinant adeno-associated virus (rAAV) serotype 9 vector to facilitate transduction of non-dividing cells. A single tail-vein injection in SBMA mice induced sustained and widespread overexpression of miR-298 in skeletal muscle and motor neurons and resulted in amelioration of the neuromuscular phenotype in the mice.

**Figure:**
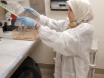


## Introduction

MiRNAs are non-coding RNAs, 21-23 nucleotides in length, that play an important role in the regulation of gene expression and control of diverse cellular and metabolic pathways.^1^ Gene expression is mainly regulated by inducing post-transcriptional gene silencing or mRNA degradation.^2^ MiRNAs are usually complementary to the 3' untranslated region (UTR) of coding genes, although binding to the 5'UTR and coding regions of the target mRNA has also been described.^3^

As the current understanding of the roles of miRNA in the pathogenesis of human diseases expands, pharmacological modulation of individual miRNAs or miRNA families is increasingly becoming a viable therapeutic option. Compared to other RNA inhibition strategies, miRNAs have many advantages: miRNAs are less toxic and less immunogenic and can be easily delivered into cells because of their small size.^4^,^5^,^6^ MiRNAs typically have many targets within cellular networks, therefore potential off-target effects and safety concerns need to be taken into account, together with efficient delivery to target tissues.

Neuromuscular diseases are acquired or inherited conditions that affect muscle and motor neurons. The targeting of drugs to skeletal muscle is an emerging area of research, where the main challenge is to achieve widespread distribution within the therapeutic window.^7^ Motor neurons are more difficult to target, mainly because drug access is precluded by the blood brain barrier.

Cell culture and mouse models of spinal and bulbar muscular atrophy (SBMA) were used in this study. SBMA is a neuromuscular disease caused by a toxic gain of function mechanism, in which both muscle and motor neurons are affected.^8^,^9^ SBMA (Kennedy's disease; OMIM #313200) is an X-linked disease, characterized by muscle weakness and atrophy, caused by CAG repeat expansion in the AR gene, which encodes an extended polyglutamine tract in the *AR* protein.^10^ No disease-modifying treatment is currently available for this disorder. The transgenic mouse model used in this study recapitulates the features of the disease, including the gender specificity, motor-neuron pathology, and progressive muscle atrophy.^11^

In this study, our efforts focused on identifying a miRNA that directly downregulates expression of the mutant AR transgene and on designing a safe and efficient mode of delivery of the miRNA to the spinal cord and skeletal muscle of our disease mouse model.

Here we identified a relatively uncharacterized miRNA, miRNA-298 (accession number MIMAT0004901),^12^ to directly reduce mutant AR expression in SBMA models. In order to achieve delivery of miR-298 to target tissues, we used a viral strategy, with recombinant adeno-associated virus serotype 9 (rAAV9). rAAV9 is capable of crossing the blood brain barrier and mediating long-term gene expression in non-dividing cells, including neurons.^13^ A single systemic administration of AAV9-miR-298 resulted in sustained expression of the miRNA, efficient transduction of muscle and motor neurons, down-regulation of AR expression, and amelioration of the disease phenotype in SBMA mice.^14^ This methodology can be used to provide miRNA or antagomirs overexpression *in vivo*.

## Protocol

All procedures were carried out in accordance with the National Institutes of Health Guide for the Care and Use of Laboratory Animals (8th ed., National Academies Press, Revised 2011), and have been approved by the NINDS Animal Care Committee.

### 1. AAV9-miRNA design strategy and miRNA selection

1. Use miRWalk predictive database to select candidate miRNAs that interact with the mRNA 3'UTR region of the target gene.^15^ NOTE: Restrict minimum seed length of miRNA sequence to at least 7 nucleotides. Select other established miRNA prediction programs (miRanda, miRDB, Targetscan) for comparative analysis. Based on these selection criteria, here, miR-185, miR-298, miR-873, and miR-877 were selected for further evaluation.
2. Retrieve the sequence of the selected miRNA from miRBase (http://www.mirbase.org/).
3. Clone the pri-mir (60- 70 nts) and its 250-300 nts flanking genomic sequence on both sides, or a mock sequence, into the appropriate AAV cis vector. NOTE: The flanking sequence is required for correct pri-mir expression and mature microRNA processing. Select a dual-promoter rAAV cis vector with an expression cassette consisting for example of a human elongation factor-1 alpha (EIF1α) promoter followed by the selected miRNA or mock sequence, and the human cytomegalovirus (CMV) promoter followed by cDNA encoding the GFP fluorescent tag. The EIFα promoter was chosen because it guarantees stable and homogeneous expression, with minimal risks of silencing. Tissue specific or conditional promoters can be used, depending on specific applications.
4. Prepare, purify, and titrate the AAV, following published protocols.^16^ NOTE: Here, cloning of the pri-mir into the AAV vector and viral production were performed by an external manufacturer.

### 2. Tail Vein Injection of AAV-miRNA Plasmid

NOTE: This step needs adjustment according to the target gene and the age and weight of the mice. Use the Institutional and Animal Care and Use Committee (IACUC) guidelines to determine the dose range, volume and best route of administration based on the age and weight of the mice. GFP fluorescence signal and miRNA expression levels in target tissues are expected to peak starting 2 weeks after the injection. The following protocol refers to male mice in early adulthood. AAV should be handled as a biohazard under Biosafety Level 1 guidelines.

1. Divide AAV-miRNA plasmid stock in 100-200 µL aliquots containing a viral load of 10^10^-10^11^ viral genomes per mL (vg/mL) using sterile phosphate buffered saline (PBS). Laboratory biohazard personal protective equipment should be used to handle the AAV solution. NOTE: Once an aliquot is thawed it can be stored at 4 °C for up to two weeks. Viral stock can be stored in -80 °C freezer.
2. Restrain the mouse in commercially available restrainers such as plastic or tapered plastic film tube of proper size.
3. Clean the surface of the tail with 70% alcohol wipes.
4. Hold a warm pad (28-30 °C) under the tail to increase vasodilation for 30 s. Alternatively, warm the animals using a red heat lamp (at a distance of 2-3 feet) or by dipping the tail into warm water.
5. **Starting from the distal portion of the tail, insert the needle (27-30 G) loaded with the viral aliquot, bevel up, 15° from the tail, into the vein. Before injection, ensure the needle is inserted correctly.**
  1. If resistance is felt during injection, or swelling appears under skin, stop the procedure. Remove the needle and re-insert the needle above the previous injection site. NOTE: Alternatively, this can be achieved by gently aspirating the needle and observing blood. Hard aspiration can collapse the tail vein. If the vein blanches during injection, the needle is inserted correctly.
6. After the injection has been completed, wait a few seconds before raising the needle.
7. Observe the injection site for bleeding. Apply moderate pressure using two fingers to the site until bleeding stops.
8. Return the animal to its cage.

### 3. Behavioral Assays

NOTE: All experiments were conducted blindly by a third party with concealment of treatments by uniquely coded vials. Order of treatments was randomized. When performing the tests described below, experimental conditions (type of room and time of the day) should be controlled to reduce variation. This test should be performed on mice older than four weeks to attain reliable results. Mouse underwent behavioral assessment once before viral injection at 5 weeks of age to obtain baseline of normal performance. After viral injection, mice were assessed once a week starting 48 h after injection, up to 40 weeks of age.

1. Bring the mouse injected with AAV to a quiet, dark room that is free of noise or other disturbances at least 30 min before starting the tests. Do not disturb the animals during this period of acclimatization.
2. Acclimatize the mouse to the new room for 30 min and then start the following behavioral assays (steps 3.3 and 3.4). Keep a gap of 10 min in between each behavioral assay.
3. **Hanging wire test** NOTE: This test monitors muscle strength and motor dysfunction.
  1. Use a padding sheet to act as a cushion. Pick up the mouse by its tail and place it on the center of the wire grid rack. Raise the rack 15-20 inches above the surface and slowly invert the screen to allow the animal to adjust its grip on the screen.
  2. Once the rack is completely inverted, start the timer, and record the time to fall. NOTE: Mice will hang with four limbs on a grid. If the wire is set too low the mice may not hang on. If mice fall off the grid before 60 s, place them back on the grid, reset the timer, and repeat this procedure up to two more tries. Register the best performance.
  3. When the time limit of 60 s is reached, return the animal back to the cage.
4. **Foot print assay** NOTE: The absorbent sheet should be a narrow runway about 70 cm long and 5 cm wide with 5 cm high walls.
  1. Hold the mouse gently by one hand and apply red ink to the front paws and blue ink to the hind paws with a brush.
  2. Place the absorbent sheet on the floor of a narrow runway.
  3. Allow the mouse to walk or run over an absorbent sheet in a straight line in the runway from one end to other.
  4. Repeat the process two more times with the mouse using a fresh absorbent sheet.
  5. Measure the distance between two consecutive steps in the forward movement. Do not include first and last few footprints where the animal is just initiating and finishing its run.

### 4. Euthanasia and Tissue Harvest

1. Use a euthanasia chamber or a bell jar. If using a bell jar, perform the procedure under a fume hood.
2. Soak cotton with isoflurane and place it in the bell jar. Use a physical separator to keep the animal from physical contact with the anesthesia material. Place the animal in the jar immediately after this step. NOTE: The bell jar should not be pre-charged with isoflurane to avoid the possibility of hypoxemia in the animals.
3. Continue isoflurane exposure until 30 s after breathing stops.
4. Immediately after removing the mouse from the jar, euthanize by cervical dislocation. NOTE: This step should take place as rapidly as possible to avoid tissue degradation.
5. **Harvest the spinal cord first and then the quadriceps skeletal muscle.**
  1. To harvest the spinal cord, expose the backside of the mouse and fix the four limbs on the side to a dissection board.
  2. Wash the site of dissection with 70% ethanol.
  3. Perform cervical dislocation using a sharp scissor.
  4. Make an incision in the skin in the median line. Remove the pelt by either cutting or careful tearing of the skin in the transverse plane, followed by pulling the pelt up and over the head. Remove the head by cutting with scissors beneath the shoulder blades and at the C1-2 region of the column (found adjacent to the base of the skull).
    1. Cut the abdominal wall musculature on the ventral side and continue laterally, one direction at a time, until the spinal column is reached.
  5. Using a sharp scissor remove vertebrae starting from the cervical spinal cord. Remove the lateral parts of the vertebrae by cutting across the vertebral arches at both sides.
  6. After removing the entire vertebrae release the spinal cord.
6. **Snap freeze the harvested tissues in pre-chilled 2-methylbutane using the following method:**
  1. Half fill a stainless steel bowl with 2-methylbutane and submerge the bottom quarter of the bowl in a container filled with liquid nitrogen.
  2. Pre-chill the 2-methylbutane in liquid nitrogen for 1-2 min.
  3. Place the skeletal muscle with forceps in the 2-methylbutane until it turns white. Immediately transfer the tissue to dry ice and then store it at -80 °C.
7. For spinal cord immunostaining, embed the tissue in paraffin blocks at room temperature before snap freezing as described.^17^
8. For biochemical analysis and miRNA quantification,^1^ place the harvested tissue in a cryovial and freeze in liquid nitrogen. Store the samples at -80 °C.

## Representative Results

A viral load of 10^11^ vg of AAV9-miR-298 was injected through a single tail-vein injection into 5 week old SBMA mice. These mice carry the human AR transgene with abnormally expanded polyglutamine tract in the AR (AR97Q) and develop signs of neuromuscular disease by 10 weeks of age (weight loss, hunched back, and muscle atrophy).^11^ Lumbar spinal cord and quadriceps muscle were harvested at 2, 4, 8 and 12 weeks after administration for miRNA quantification, biochemical assay and immunohistochemistry (Figure 1). Administration of the treatment and the subsequent analyses were performed by blinded investigators.

qRT-PCR analysis showed miR-298 expression in the skeletal muscle and the spinal cord two weeks and four weeks after treatment respectively, with peak expression levels at 8 weeks in the skeletal muscle and 12 weeks in the spinal cord after the injection (Figure 2). Using a microscope (Axiovert 100 M), green fluorescence signal was detected in muscle tissue and in spinal motor neurons by co-localization of GFP and the motor neuron marker choline acetyltransferase (ChAT) 10 weeks after treatment, when the mice start to show disease manifestations (Figure 2).

Using the same dose regimen, a cohort of SBMA mice was randomized to receive either miR-298 or mock at 7 weeks of age via tail vein injection for biochemical analyses and functional characterization. Injection was followed by weekly weight and behavioral assessment up to 40 weeks of age. qRT-PCR analysis showed that miR-298 treatment reduces the levels of mutant AR in affected tissues (Figure 3), and increased body weight and improved motor performance (Figure 4) starting at 10 weeks after the injection.

**Figure:**
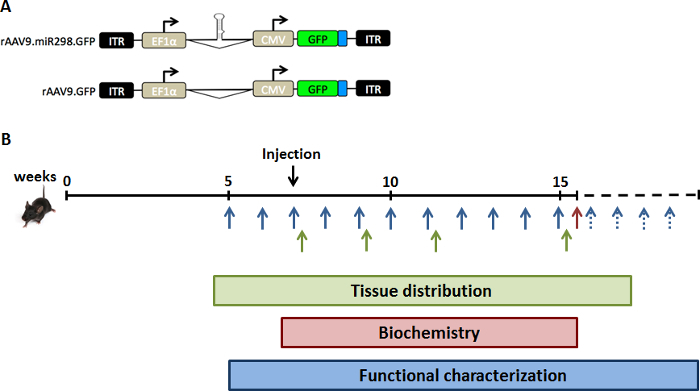

**Figure 1: Schematic of study design.** To increase expression levels of miR-298 *in vivo, *we injected SBMA mice with (**A**) the dual promoter AAV vector plasmid expressing GFP and either miR-298 or mock. (**B**) Mice were injected via a single tail-vain injection at 7 weeks of age (pre-symptomatic stage). Spinal cord and quadriceps muscle were collected from a cohort of SBMA mice at different time points for tissue distribution analysis (green). A cohort of SBMA mice was treated and sacrificed at 16 weeks of age for biochemical analyses (red). Weight and behavioral assays were performed weekly up to 40 weeks of age for functional characterization (blue). Please click here to view a larger version of this figure.

**Figure:**
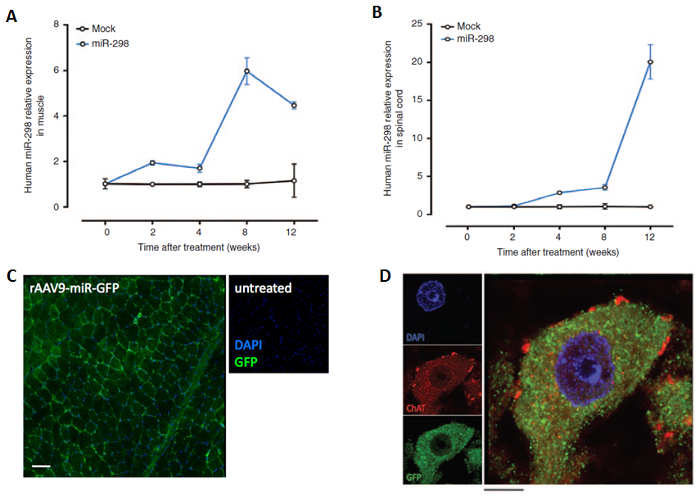

**Figure 2: AAV9-miR-298 delivery in mice.** Using the method described here, mice received either AAV9-miR-298-GFP or AAV9-mock-GFP through intravenous injection. Total miRNA was collected from lumbar spinal cord and quadriceps muscle at 2,4,8 and 12 weeks after injection. qRT-PCR was performed to estimate expression level of miR-298 in (**A**) quadriceps muscle (n = 5) (**B**) lumbar spinal cord (n = 5, P <0.01). All data are reported as means ± standard error mean. The widespread transduction of the AAV vector in tissues harvested at 10 weeks after treatment was confirmed by localization of staining for GFP in the (**C**) quadriceps muscle (original magnification, 10X. Scale bar = 100 µm) and (**D**) motor neurons in lumbar spinal cord (original magnification, 40X. Scale bar = 10 µm. GFP (green), ChAT (red) and DAPI (blue). The figure has been modified from doi: 10.1038/mt.2016.13.^14^ Please click here to view a larger version of this figure.

**Figure:**
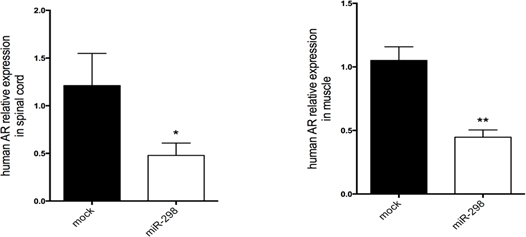

**Figure 3: MiRNA-298 over-expression downregulates mutant AR in spinal cord and quadriceps muscle in mice.** qRT-PCR was performed to estimate expression levels of AR mRNA in (**A**) lumbar spinal cord and (**B**) quadriceps muscle treated with AAV9-miR-298-GFP or AAV9-mock-GFP. Transcript levels were normalized to snoRNA202 (n = 5 per treatment). *P <0.05, **P <0.01. All data are reported as means ± standard errors. The figure has been modified from doi: 10.1038/mt.2016.13.^14^

**Figure:**
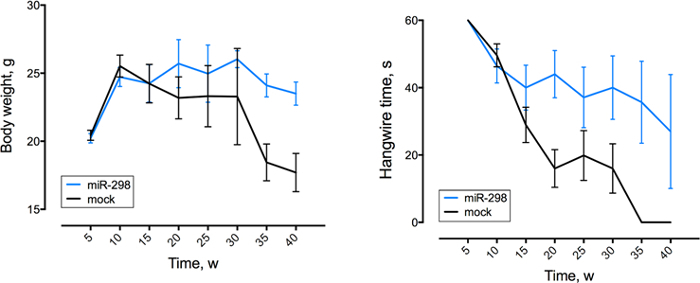

**Figure 4: MiR-298 over-expression improves motor function and reduces weight loss.** Behavioral assessment was performed once a week (w), between week 5 to 40. Body weight (left) and hanging wire (right) performance of mice (n = 15 per group). All data are reported as means ± standard errors. The figure has been modified from doi: 10.1038/mt.2016.13.^14^

## Discussion

Here we demonstrate a highly efficient and accessible methodology to select and deliver via tail injection a miRNA using rAAV9 as a viral vector to target skeletal muscle and motor neurons in mice.^14^ Compared to other RNAi strategies, miRNAs are less toxic and less immunogenic.^18^ In addition, their small size make them well suited to the limited packaging capacity of viral vectors.^2^ Computational algorithms and prediction tools allow the identification of putative miRNA-mRNA targets. Once identified, the effects of miRNA on target gene expression must be verified. A common approach is to overexpress a given miRNA *in vitro* and detect target protein expression levels using western analysis.^14^,^19^

Various studies have used viral and non-viral strategies for delivering miRNAs.^13^ Here we used adeno-associated virus (AAV) as a gene delivery tool. Compared to other viral vectors, AAV elicits low immunogenicity, allows long-term gene transfer, and has a broad spectrum of tropism in dividing and non-dividing cells.^18^ This delivery method can also circumvent the need for chemical modification that may affect functionality and specificity of the RNA molecule. There are several serotypes of AAV, which are mostly determined by the composition of the capsid proteins. These serotypes differ in their tropism and transduce different cell types. It is necessary to select the correct serotype when considering the target tissue. We selected rAAV9, due to its high transduction efficiency in the central nervous system and skeletal muscle following peripheral administration^19^,^20^. This approach has shown greater transduction efficacy in neonatal animals compared to adult animals, likely due to differences in extracellular matrix composition, neuron-to-glia ratio, and maturity of the blood-brain-barrier.^21^,^22^

An important step in this protocol is the design of the expression vector. Compared with bicistronic vectors, which are hampered by lower expression of the second gene compared with the first gene next to the promoter, the dual promoter vector allows a back-to-back configuration yielding high expression of both the miRNA and EGFP, thus allowing localization of the miRNA in mouse tissue by immunofluorescence.

Intravenous injection of AAV9 resulted in high efficiency and homogenous transduction in the target tissues, skeletal muscle, and motor neurons. This route of injection allows the administered dose to reach the systemic circulation and cross the brain blood barrier, which is important for therapies targeting the central nervous system. Furthermore, it is a non-invasive method for delivery to the CNS. MiR-298 expression increased in the spinal cord and muscle after 2-4 weeks with a single peripheral injection at 5 weeks of age. When using single-stranded genome AAV vectors, the *de novo* synthesis of the second DNA strand may account for the delayed transduction.^23^

Levels of human miR-298 were undetectable in treated mice 20 weeks after a single administration, suggesting that for chronic diseases, multiple injections may be required to reach a therapeutic benefit. However, miRNA degradation over time can be limiting when a lifelong repeated administration is required. In addition, adaptive immunity to AAV vectors can form another barrier to a successful gene delivery.^24^ Thus generation of AAV vectors that are immunologically inert is crucial for repeated administration and achieving long-term effects with this promising delivery system.^25^

A limitation on the use of miRNA as a therapeutic strategy is the risk of off-target effects. MiRNAs may interact through incomplementary base pairing with other gene transcripts, which poses a safety risk with this approach. Improved designing of the RNAi sequences, including use of non-canonical miRNAs, such as mirtrons, which bypass the micro-processor complex and extensive long-term safety and tolerability studies are critical before translating this strategy into a safe and effective therapy.^26^,^27^,^28^

The method described here was originally developed for miRNA overexpression, but it can also be utilized for miRNA inhibition therapy using antagomirs.

## Disclosures

The authors have nothing to disclose.
